# Heat Capacities of Polyethylene IV. High Molecular Weight Linear Polyethylene

**DOI:** 10.6028/jres.080A.009

**Published:** 1976-02-01

**Authors:** Shu-Sing Chang

**Affiliations:** Institute for Materials Research, National Bureau of Standards, Washington, D.C. 20234

**Keywords:** Glass transition, heat capacity, high molecular weight linear polyethylene, polyethylene, temperature drift, thermodynamic properties

## Abstract

A high molecular weight linear polyethylene sample has been studied by adiabatic calorimetry from 10 to 380 K. Two broad temperature regions of unusual spontaneous temperature drift have been observed. The phenomena occurring around 240 K are similar to that observed in other polyethylene samples studied in this series, and are presumed to be caused by the relaxational processes in the amorphous phase. The weak exothermic behavior occurring around 160 K is presumed to be caused by the stabilization of the quenched sample.

## 1. Introduction

The heat capacity behavior of a sample of high molecular weight linear polyethylene has been studied recently by Beatty and Karasz [1, 2].^1^ High molecular weight polyethylene is more difficult to crystallize than lower molecular weight polyethylene. Increased amorphous content should provide a stronger indication of the existence of a glass transition in partially crystalline linear polyethylene. A region of rather abrupt change in the heat capacity differences between that high molecular weight sample and a linear polyethylene sample studied by Dainton et al. [3] was noted. For the quenched high molecular weight sample, there was an 8 percent increase in the heat capacity occurring in the temperature region 130 to 190 K. For the annealed sample, there was about 5 percent increase. At higher temperatures a bend in the heat capacity curve was noted similar to Dainton’s data. The region near 150 K was assigned as the glass transition temperature of linear polyethylene. Spontaneous temperature drifts under adiabatic conditions, indicative of relaxational processes, were not noted in detail.

A similar high molecular weight linear polyethylene sample from the same manufacturer was studied in this laboratory in the temperature range from 10 to 380 K. Detailed spontaneous temperature drifts were carefully observed on the sample under various thermal treatments. The results and conclusions from this study are somewhat different from the previously mentioned literature.

## 2. Experimental Section

### 2.1. Calorimetry

Heat capacity measurements on the high molecular weight linear polyethylene sample were made with the same vacuum adiabatic calorimeter [4] that was used in the previous studies [5, 6] on polyethylene samples derived from Standard Reference Materials 1475 and 1476.

### 2.2. Materials

A sample of high molecular weight polyethylene was furnished by Dr. R. J. Schaffhauser of the Plastics Division, Allied Chemical Corp.,^2^ bearing the designation 260–100. This resin was at one time also designated as AC8X, as was Beatty’s sample [2]. It was produced by Ziegler-type vapor phase polymerization. Its molecular weight is estimated by the manufacturer as 8–13 × 10^6^. It has an ash content of 0.04 percent and contains no additives. The molecular weights are estimated in the order of 2.7–3 × 10^6^ by both viscometry and light scattering measurements from the Polymer Characterization Section of the National Bureau of Standards. Whether the discrepancy in the molecular weights is caused by the degradation of the polymer at high temperatures or by differences in the procedures to estimate the molecular weight was not determined.

The sample was received in the form of a powder. Attempts to fuse the powder together under its own weight in vacuum at 150 °C were not successful due to the high viscosity of the melt. The heating produced a sintered product. In order to avoid degradation of the high molecular weight material at high pressure and temperature, molding was not tried.

The material was pressed into pellets of about 1.27 cm in diameter and about 1 cm in height in a hydraulic press at room temperature. 73.891 g (mass in vacuum) of the material was loaded in the sample container for at 3.8 cm Hg (5.1 kPa) at room temperature was sealed in to improve thermal conduction within the sample container.

## 3. Results

**Figure 1:**
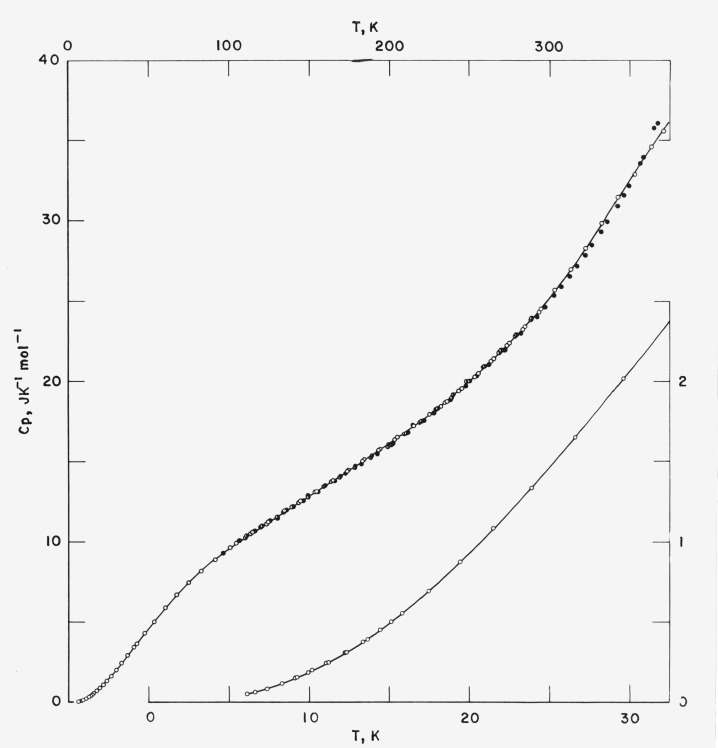
Heat capacity of high molecular weight linear polyethylene. ◑ Annealed at <300 K, ● annealed at 370 K, ○ quenched.

The results of the heat capacity measurements are tabulated in table 1 and shown graphically in figure 1. Table 1 is separated into various sections according to the thermal treatment the sample received. Within the section, the data are arranged in the order of increasing initial temperature of a series of heat capacity measurements. The series are numbered in chronological sequence throughout table 1 in order to facilitate the tracing of the thermal history of the sample.

Quenched samples were produced by admitting helium gas to the cryostat while the assembly was submerged in liquid nitrogen. A cooling rate in the order of 4–5 K min^−1^ was achieved. Slow-cooling (rate annealing) rates of 0.5–1 K h^−1^ were often used in conjunction with isothermal annealing (soak annealing) at a particular temperature for a period of days.

The temperature increment for a heat capacity determination may be inferred from the differences in the mean temperatures of the adjacent determinations within the series. Curvature corrections have been added to correct for the effect of the finite temperature increment of a determination. The precision of the measurement above 25 K is in the order of 0.05 percent. Below 25 K, the precision gradually changes to about 1 percent at 5 K. The accuracy over most of the temperature range of the measurement is comparable to the precision as indicated by heat capacity measurements on a Calorimetry Conference standard sample of sapphire [4].

Smoothed heat capacity values representing those of a quenched (5 K min^−1^) high molecular weight linear polyethylene are listed in table 2. Enthalpy and entropy increments referring to the zero point enthalpy and the residual entropy of the sample are also listed. Since the partially crystalline sample is expected to have undetermined residual entropies at 0 K, Gibbs free energy is not given in table 2.

## 4. Discussion

The crystallinity of 45 percent for this powdery sample was estimated from heat of fusion measurements using a dynamic scanning calorimeter. Heat of fusion of a pressure crystallized linear polyethylene (96% crystallinity) [6] was used as the reference.

Although the high molecular weight sample has lower crystallinity than the SRM 1475 in the condition as received (71% crystallinity) [5], it shows a somewhat lower heat capacity over the temperature range 90 to 310 K. Only at lower and higher temperatures does this sample exhibit the expected higher heat capacity. The heat capacity of this sample is very similar to that of a sample of SRM 1475 slowly cooled from the melt (88% crystallinity) [6] in the temperature range 100 to 220 K.

**Figure 2:**
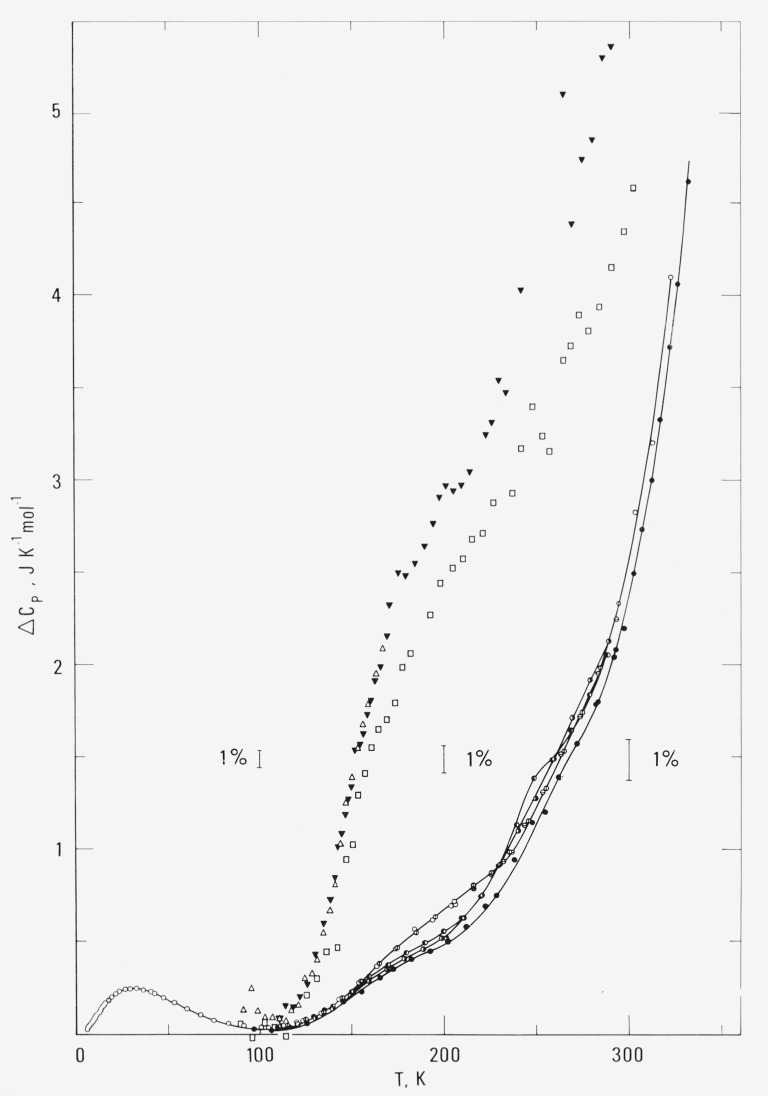
Heat capacity differences of high molecular weight linear polyethylene. This research: ◐ and ◑ annealed at < 300 K; ● annealed at 370 K; ○, ⦸ and ⊖ quenched. Beatty [2]: △ run 1; ▼ run 2; ☐ run 3.

In a plot of heat capacity differences, figure 2, between the heat capacity of this sample and that extrapolated for crystalline linear polyethylene [6], the changes in the 150 and 240 K regions are not as large as that for the 71 percent crystallinity sample. Beatty’s data [2] are also included in figure 2. A relatively rapid heat capacity increase over that of the crystalline linear polyethylene occurs in the temperature region of 120 to 170 K. Above 200 K, the scattering of the data causes difficulties in the conclusion of either the existence or nonexistence of a subtle heat capacity change. DTA or DSC studies on some other high molecular weight polyethylene samples suggested the occurrence of a step change in the heat capacity around 150 K [7, 8] and indicated a surprisingly constant heat capacity difference between the high molecular weight sample and a high crystallinity sample [8].

The lack of a strong glass-like feature in the heat capacity behavior of this high molecular weight material is in parallel to a recent thermal expansivity study on the same materials. Low temperature thermal expansions of polyethylene samples have been studied on strips of compression molded thin films [9]. The samples included the two Standard Reference Materials 1475 and 1476, used in a previous calorimetric study [5], and the high molecular weight sample used in the present calorimetric work. In all three samples, double peaks in the derivatives of the thermal expansion coefficients at about 110 and 150 K were observed. These peaks are stronger in linear polyethylene than in branched polyethylene. Weaker peaks were observed in the temperature range 180 to 200 K. Above 200 K, there were strong peaks in the branched polyethylene, a weak one in the high molecular weight sample and only a monotonic increase in the ordinary linear polyethylene.

Although the appearance of a discontinuity in the heat capacity behavior is perhaps a necessary condition for a glass transition, it is not a sufficient condition. A broad distribution in the relaxation times may cause the glass transformation to occur over a wide temperature range, thus making a weak heat capacity discontinuity in partially crystalline material even more difficult to assess. Since the glass transition is kinetic in nature, relaxational phenomena should also be observed in the glass transition region. One highly sensitive method to detect the thermal relaxation is the method of observing the spontaneous temperature changes of the sample under adiabatic conditions [10]. The sign and the magnitude of the temperature drift as a function of time and temperature may be correlated with different thermal treatments and histories. Both the discontinuity in the heat capacity behavior and the adiabatic temperature drift are commonly observed in glass transition regions of various materials and also in glass-like transitions in crystals [11].

**Figure 3:**
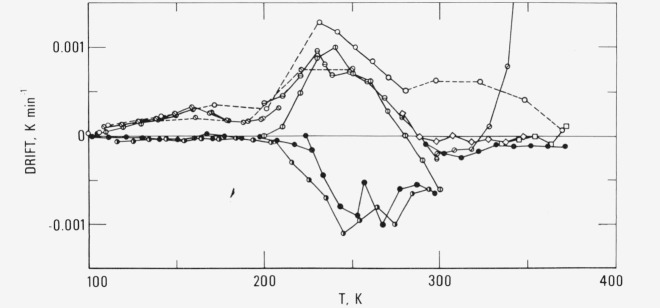
Spontaneous adiabatic temperature drift of high molecular weight linear polyethylene. Open circles and squares (quenched): ⦶ Ser. III and IV, ⊖ V, ⦸ VIII, ⊘ XII, ◇ XIII, ☐ XIV. ○ before Ser. XV and ⊗ before Ser. IX, drift observations only. Filled or half-filled circules (annealed): ◑ I and II, ● XV–XVII.

The drift observations are shown in figure 3. These are the drift rates observed at 20 minutes after the heater energy has been turned off. Because of the geometric arrangement of various components in the sample container [4], the temperature of the thermometer and the heater is slightly higher than that of the sample during the heating period. Therefore, when the energy is turned off, there is an exponential decay of the temperature of the thermometer until a thermal equilibrium is reached between the sample and the sample container assembly. The decay constant depends on the construction of the sample container, the thermal conductivity of the sample and the amount of helium gas sealed in the sample container. The decay constant is about 50 s for this sample container [12] at high temperatures and less at lower temperatures. Therefore up to 10 to 15 minutes may be required for the sample to reach a temperature distribution which is uniform to within 10^−4^ K.

The drift behavior in the 240 K region for the high molecular weight material occurs in similar temperature range and magnitude as that observed for the as-received SRM 1475 [6, 10] and is presumed to be caused by the relaxational behavior in the amorphous phase. For the quenched sample, i.e., cooling rate is greater than the rate of heat capacity determination of 5–10 K h^−1^, the drift rate is generally positive (exothermic) with a broad peak around 230 K. There is also a weaker peak at around 160 K. When the sample was heated to 370 K for the first time, large exothermic behavior showed up above 330 K, indicating the onset of further crystallization processes. For the sample either slow-cooled continuously to 100 K or annealed at 225 and 140 K, there is an endothermic peak only around 245 K. No endothermic behavior was observed at temperatures below 210 K for the sample either slow cooled or annealed. Thus the weaker exothermic peak occurring around 160 K for quenched samples is presumed due to some stabilization processes such as the relief of strain.

**Figure 4:**
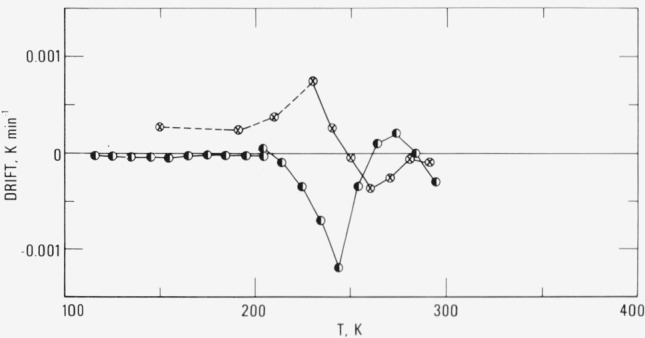
Spontaneous adiabatic temperature drift of high molecular weight linear polyethylene. ⊗ Annealed at 250 K and then quenched from 250 K (before Ser. IX). ◐ Quenched from 360 K and then annealed at 225 K and 143 K (Ser. VI and VII).

In the broad temperature region near 240 K, the drift behavior as observed in figure 3 and in other linear polyethylene samples [6, 10] seems to indicate the existence of double peaks. Special thermal treatments were performed in attempts to resolve these peaks. Figure 4 shows the results of the drift observations for two combinations of quenching, annealing and slow cooling treatments. The sample was first annealed at 250 K for 1 day and then quenched from 250 K. Upon heating the sample, it showed exothermic behavior, similar to that of a quenched sample, at temperatures below 250 K. Above 250 K, endothermic effects similar to an annealed sample were seen.

The sample was quickly cooled (5 K min^−1^) to 225 K and held there for 4 days, until the drift decreased from an initial value of greater than 1 mK min^−1^ to less than 0.05 mK min^−1^. It was then slowly cooled (2.5 K h^−1^) to 143 K and held there for 2 days. An endothermic peak was observed at about 245 K. Above 245 K, a small exothermic peak appeared near 270 K. Apparently annealing at 225 K did not relax the configurations associated with the higher temperature peak.

The two regions around 240 and 270 K may be caused by different types of amorphous relaxation mechanisms, such as from the loose ends of the polymer molecules or from the molecular segments with both ends confined in crystalline regions. However, the mechanisms responsible for the relaxational behavior observed here have not been determined.

## Figures and Tables

**Table 1:** Heat capacity data of high molecular weight linear polyethylene

*T, K*	*C_p_*, J k^−1^ mol^−1^	*T, K*	*C_p_*, J K^−1^ mol^−1^

Quenched			

SERIES X		SERIES III	
6.13	0.0507	104.82	9.895
6.62	.0623	114.82	10.59
7.38	.0843	124.63	11.24
8.33	.1170	134.58	11.88
9.26	.1558	144.57	12.51
10.18	.1982	154.63	13.16
11.18	.2484	164.68	13.81
12.33	.3130	174.62	14.46
13.64	.3954	184.56	15.11
15.13	.5016	194.64	15.78
	
SERIES IX		SERIES VIII	
	
9.11	0.1500	113.50	10.50
9.96	.1875	123.38	11.16
11.06	.2445	133.50	11.81
12.27	.3106	143.76	12.46
13.37	.3773	153.86	13.11
14.48	.4536	163.71	13.74
15.80	.5549	173.57	14.39
17.50	.6963	183.45	15.06
19.45	.8758	193.35	15.69
21.51	1.082	203.39	16.36
23.87	1.335		
26.59	1.649		
29.63	2.015		
32.96	2.437		
36.62	2.916		
40.68	3.453		
	
SERIES XI		SERIES IV	
	
42.32	3.663	205.32	16.51
47.20	4.296	215.33	17.21
53.44	5.085	225.29	17.92
60.36	5.906	235.29	18.69
67.54	6.690	245.31	19.55
74.97	7.436	255.21	20.44
82.91	8.171	265.11	21.38
91.40	8.882	274.99	22.37
100.88	9.609	284.83	23.41
		294.73	24.55
	
SERIES V		SERIES XII	
	
205.41	16.50	303.29	25.70
215.44	17.19	313.08	26.96
225.40	17.92	322.98	28.27
232.41	18.45	332.98	29.80
236.45	18.77	342.96	31.43
243.46	19.40	353.02	32.84
253.42	20.29	363.28	34.56
263.36	21.23	370.79	35.52
273.38	22.21		
283.37	23.26		
293.33	24.35		

Slow-Cooled from 295 K			

SERIES I		SERIES II	
	
111.21	10.35	201.20	16.06
120.85	10.98	210.86	16.75
130.04	11.57	220.65	17.49
139.42	12.16	230.44	18.30
149.33	12.79	240.24	19.14
159.23	13.41	250.03	20.02
169.02	14.02	259.82	20.95
178.84	14.64	269.70	21.92
188.70	15.26	279.57	22.92
198.69	15.91	289.33	23.96
	
Quenched and then annealed at 225 K, and 143 K			
	
SERIES VI		SERIES VII	
	
110.07	10.27	209.60	16.67
120.01	10.94	219.52	17.42
129.92	11.57	229.35	18.23
139.87	12.20	239.08	19.10
149.65	12.83	248.78	20.03
159.42	13.44	258.62	20.84
169.42	14.07	268.58	21.76
179.44	14.70	278.62	22.76
189.48	15.34	288.71	23.84
199.55	16.00		

Annealed at 370 K			

SERIES XIII		SERIES XIV	
	
283.27	23.10	340.49	30.54
292.99	24.16	349.78	32.17
302.85	25.33	358.71	33.95
312.75	26.54	367.28	36.05
322.67	27.87		
332.59	29.29		
342.49	30.88		

Slow-Cooled from 370 K			

SERIES XV		SERIES XVII	
	
96.80	9.29	228.40	18.00
106.83		238.11	18.84
116.47	10.69	247.90	19.72
126.09	11.31	254.90	20.29
135.86	11.96	262.09	21.01
145.80	12.55	272.14	21.97
155.72	13.17	282.08	22.99
165.63	13.79	292.09	24.05

SERIES XVI		SERIES XVIII	
	
172.39	14.21	297.52	24.63
182.23	14.83	307.32	25.90
192.21	15.45	317.06	27.14
202.23	16.10	326.91	28.46
212.18	16.79	336.84	29.93
222.06	17.52	346.75	31.57
		356.51	33.45
		366.15	35.72

**Table 2:** Thermodynamic properties of high molecular weight Linear Polyethylene (–CH_2_– = 14.027)

*T*, *K*	*C_p_*, J K^−1^ mol^−1^	H–H_0_, J mol^−1^	S – S_0_, J K^−1^ mol^−1^
5	0.037	0.047	0.012
10	.190	.527	.073
15	.492	2.175	.203
20	.929	5.677	.401
25	1.463	11.63	.664
30	2.062	20.41	.983
35	2.702	32.31	1.349
40	3.360	47.47	1.752
45	4.016	65.91	2.186
50	4.656	87.60	2.642
60	5.862	140.3	3.600
70	6.946	204.4	4.586
80	7.909	278.8	5.578
90	8.769	362.3	6.560
100	9.545	453.9	7.525
110	10.26	553.0	8.469
120	10.94	659.0	9.391
130	11.59	771.7	10.29
140	12.22	890.7	11.17
150	12.86	1016.	12.04
160	13.50	1148.	12.89
170	14.15	1286.	13.73
180	14.81	1431.	14.55
190	15.47	1582.	15.37
200	16.14	1740.	16.18
210	16.82	1905.	16.99
220	17.52	2077.	17.79
230	18.27	2256.	18.58
240	19.09	2443.	19.38
250	19.96	2638.	20.17
260	20.90	2842.	20.97
270	21.87	3056.	21.78
280	22.89	3280.	22.59
290	24.00	3514.	23.42
300	25.20	3760.	24.25
310	26.51	4019.	25.10
320	27.90	4291.	25.96
330	29.38	4577.	26.84
340	30.91	4877	27.74
350	32.46	5194.	28.66
360	34.00	5526.	29.60
370	35.44	5877.	30.55
380	36.76	6248.	31.51
273.15	22.18	3125.	22.04
298.15	24.97	3714.	24.10

## Footnotes

1 Figures in brackets indicate the literature references at the end of this paper.

2 Commercial materials are identified in this paper to adequately specify the experimental procedure. Such identification does not imply recommendation or endorsement by the National Bureau of Standards.
